# Clinical parameters associated with metachronous recurrence after curative endoscopic submucosal dissection for early gastric cancer

**DOI:** 10.3389/fmed.2026.1965395

**Published:** 2026-10-23

**Authors:** Moon Kyung Joo, Seung Han Kim, Won Shik Kim, Beom Jae Lee

**Affiliations:** Division of Gastroenterology, Department of Internal Medicine, Korea University Guro Hospital, Korea University College of Medicine, Seoul, Republic of Korea

**Keywords:** early gastric cancer, endoscopic submucosal dissection, metachronous cancer, metachronous neoplasm, surveillance

## Abstract

Although endoscopic submucosal dissection (ESD) is an established treatment for early gastric cancer (EGC), metachronous recurrence remains an important clinical issue during long-term follow-up. We evaluated clinical parameters associated with metachronous recurrence after curative ESD using the longitudinal cohort. This retrospective observational study included 1,200 patients who achieved curative resection of EGC and were followed for at least 12 months. During follow-up, 185 patients (15.4%) developed metachronous neoplasms, including 102 patients with metachronous cancer and 83 with metachronous adenoma only. The primary analysis used a Cox proportional hazards model with time from ESD to first metachronous neoplasm or censoring. In the adjusted full-cohort model, older age [hazard ratio (HR): 1.035 per year, 95% confidence interval (CI): 1.015–1.055, *P* < 0.001] and persistent *Helicobacter pylori* (*H. pylori*) infection (HR: 2.391, 95% CI: 1.398–4.088, *P* = 0.001) were independently associated with a higher hazard of metachronous neoplasm, whereas diabetes mellitus showed a borderline association (HR: 1.391, 95% CI: 0.988–1.958, *P* = 0.058). The estimated cumulative probability of metachronous neoplasm was 7.6% at 3 years and 12.3% at 5 years. Secondary exploratory analyses using patients who remained recurrence-free for ≥10 years as controls showed associations of age and diabetes mellitus with overall metachronous neoplasm, while age and diabetes mellitus were associated with metachronous cancer and age and lower serum albumin with metachronous adenoma. In conclusion, older age and persistent *H. pylori* infection were independently associated with metachronous neoplasm in the full cohort. These findings support continued surveillance after curative ESD.

## 1 Introduction

Endoscopic submucosal dissection (ESD) has become a standard treatment for early gastric cancer (EGC) that fulfills criteria for curative endoscopic resection because it enables *en bloc* resection while preserving the stomach (1–4). Compared with surgical resection, ESD offers the advantages of organ preservation, lower procedural invasiveness, and maintenance of postoperative quality of life, while still providing excellent oncologic outcomes in appropriately selected patients (5, 6). For these reasons, ESD is now widely accepted as an effective treatment modality for EGC with negligible risk of lymph node metastasis. Despite these advantages, preservation of the stomach after ESD inevitably leaves the background gastric mucosa at risk for subsequent neoplastic change. Unlike gastrectomy, ESD does not remove the field of carcinogenic mucosal alteration, including chronic atrophic gastritis, intestinal metaplasia, and persistent inflammation. As a result, metachronous neoplasm remains one of the most important long-term limitations of endoscopic treatment (7, 8). Identifying factors associated with metachronous recurrence is therefore clinically important for understanding long-term recurrence patterns after curative ESD.

Previous studies have suggested that older age, persistent *Helicobacter pylori* (*H. pylori*) infection, severe atrophic gastritis, and synchronous neoplasms are associated with a higher risk of metachronous recurrence after endoscopic resection for EGC (8–10). However, these established risk factors do not fully explain the heterogeneity of long-term recurrence risk observed in clinical practice. In particular, there is growing interest in whether host-related systemic factors, such as inflammatory or nutritional status, may influence the development of metachronous lesions in the preserved stomach. For example, the neutrophil-to-lymphocyte ratio (NLR) is a simple inflammatory marker derived from routine laboratory testing and has been reported to reflect the balance between protumor inflammatory response and antitumor immune activity (11, 12). Likewise, serum albumin is widely used as an indicator of both nutritional condition and chronic inflammatory burden (13). These parameters have been investigated in various malignancies, but their association with metachronous recurrence after curative ESD for EGC has not been sufficiently clarified.

In our previous study focusing on clinical characteristics and outcomes of younger patients (≤50 years) with EGC undergoing ESD, age >50 years and severe atrophic gastritis were associated with metachronous gastric neoplasm during long-term follow-up after curative ESD, supporting the importance of host and mucosal background factors in recurrence risk (10). The current study extends that line of investigation by evaluating detailed baseline clinical and laboratory parameters in a time-to-event framework using the complete eligible cohort. Therefore, the primary aim of the present study was to identify baseline clinical, laboratory, and clinicopathologic factors associated with time to first metachronous neoplasm after curative ESD for EGC. Comparisons of metachronous neoplasm group with long-term recurrence-free controls were retained as secondary exploratory analyses.

## 2 Materials and methods

### 2.1 Study design and patients

This retrospective observational study included patients who underwent ESD for EGC at a tertiary university hospital in Seoul, Korea, between 2006 and 2023. Among patients who achieved curative resection after ESD, those who were followed for at least 12 months were eligible. The primary time-to-event analysis included the entire eligible cohort. Follow-up time was calculated from index ESD to the first metachronous gastric neoplasm or to the last documented recurrence-free follow-up. For secondary exploratory case-control analyses, patients who developed metachronous gastric neoplasms were compared with a selected group of patients who remained recurrence-free for at least 120 months after curative ESD; recurrence-free patients with shorter follow-up were not included in these secondary comparisons (Figure 1).

**Figure 1:**
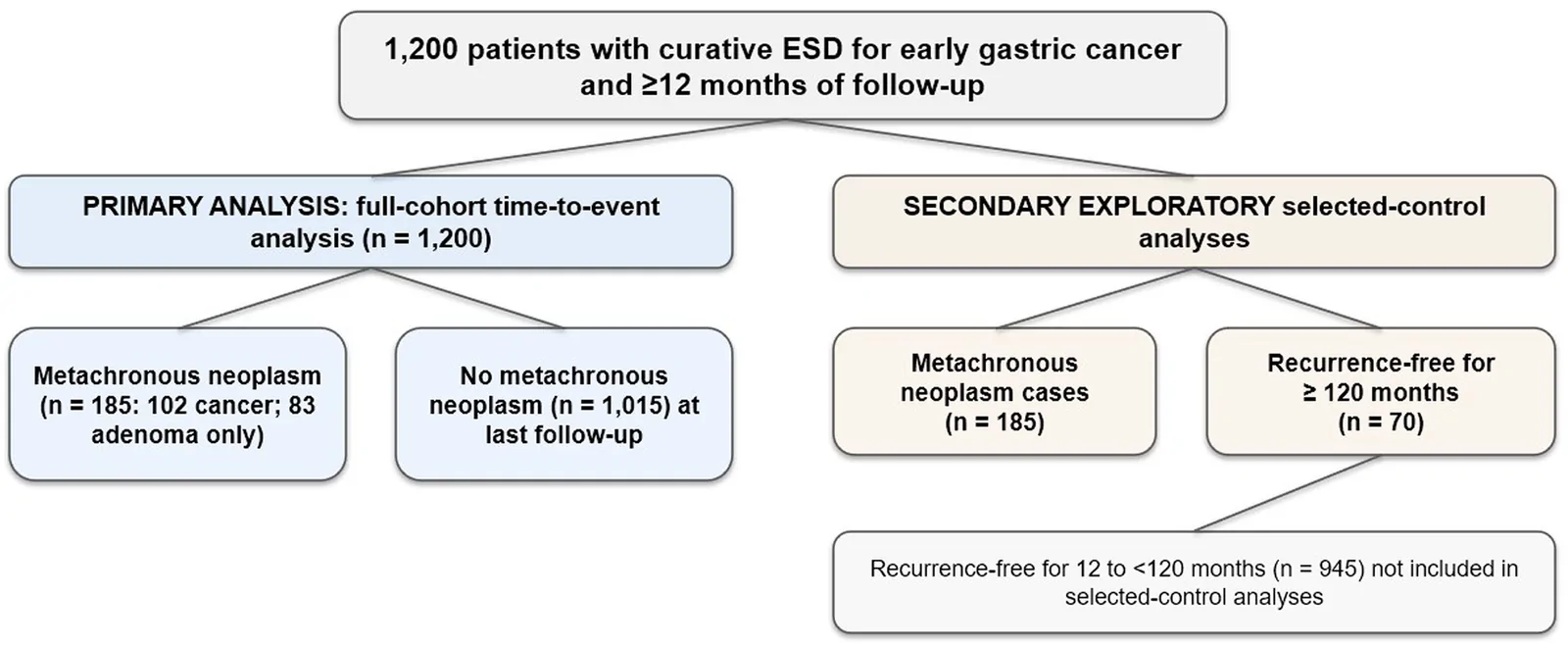
A flow diagram of patient enrollment. All 1,200 eligible patients contributed to the primary analysis; the 185-vs.-70 comparison was retained only as a secondary exploratory analysis.

### 2.2 ESD procedure and follow-up

All ESD procedures were performed using standard techniques by experienced endoscopists at our institution. In our previous ESD cohort, procedures were conducted by endoscopists with substantial ESD experience using standard ESD techniques including lesion border delineation with image-enhanced endoscopy, submucosal injection, circumferential incision, and submucosal dissection, followed by proton pump inhibitor treatment for iatrogenic ulcer healing. Follow-up endoscopy after index ESD was scheduled at 3 and 6 months and then at 12-month intervals thereafter, with cross-sectional imaging performed during long-term surveillance. The present study was conducted within the same institutional ESD and surveillance framework.

### 2.3 Data collection and variables

Demographic and clinical variables, including age, sex, family history of gastric cancer, smoking status, and comorbidities, were reviewed from the medical records. Laboratory parameters obtained at baseline included white blood cell count, neutrophil count, lymphocyte count, platelet count, NLR, platelet-to-lymphocyte ratio (PLR), erythrocyte sedimentation rate, serum albumin, prognostic nutritional index (PNI), total cholesterol, high-density lipoprotein cholesterol, low-density lipoprotein cholesterol, triglyceride, triglyceride-glucose index, serum creatinine, C-reactive protein (CRP), and fasting glucose. Clinicopathologic variables included lesion location, lesion shape, ulcer presence, tumor size, degree of endoscopic atrophy, post-ESD histologic diagnosis, differentiation type, Lauren classification, depth of invasion, indication category, *H. pylori* infection status, synchronous neoplasm, and follow-up duration. The primary Cox model was performed using complete cases for the covariates included in that model; 1,155 of 1,200 patients (180 events) had complete data for all nine covariates.

### 2.4 Definitions

Metachronous neoplasm was defined as a newly developed gastric adenoma or cancer detected at a site different from the index lesion at least 12 months after curative ESD during follow-up. Curative resection was defined on the basis of pathological findings after ESD, according to accepted criteria regarding tumor size, histologic differentiation, invasion depth, ulcer status, resection margin status, and absence of lymphovascular invasion. As in previous studies, pathologic outcomes fulfilling the absolute or expanded indication criteria were considered curative resection (14). Atrophic gastritis was classified endoscopically according to the Kimura-Takemoto classification and grouped as C1-2, C3-O1, and O2-3 (15). *H. pylori* positivity was determined by histology or rapid urease test. Persistent H. pylori infection referred to infection confirmed at the time of ESD that either failed eradication therapy or remained untreated through the last follow-up.

NLR was calculated as the absolute neutrophil count divided by the absolute lymphocyte count (16). PNI was calculated using the conventional formula: 10 × serum albumin (g/dL) + 0.005 × peripheral lymphocyte count (/mm^3^) (17). The triglyceride-glucose (TyG) index was calculated as ln [fasting triglyceride (mg/dL) × fasting glucose (mg/dL)/2], as previously described (18).

### 2.5 Statistical analysis

Baseline characteristics of the entire eligible cohort are summarized descriptively without hypothesis testing according to eventual recurrence status. For the secondary selected-control analyses, normally distributed continuous variables are presented as mean ± SD and were compared using the independent-samples t test; skewed variables are presented as median [interquartile range] and were compared using the Mann–Whitney *U*-test. Categorical variables were compared using the chi-square or Fisher's exact test, as appropriate. The primary analysis used Cox proportional hazards regression in the complete cohort of 1,200 patients, with time from index ESD to first metachronous neoplasm as the event time and recurrence-free follow-up time as the censoring time. Covariates for the adjusted model were selected *a priori* on clinical grounds to represent major host, inflammatory or nutritional, mucosal, histologic, and infection-related domains: age, sex, diabetes mellitus, NLR, serum albumin, severe endoscopic atrophy, histologic differentiation, persistent *H. pylori* infection, and synchronous neoplasm. Fasting glucose was not entered together with diabetes mellitus to avoid clinically overlapping metabolic variables. The adjusted model included 1,155 complete cases and 180 events for nine fitted covariate parameters (20 events per parameter). Hazard ratios (HRs) and 95% CIs were calculated. The proportional hazards assumption was assessed using Schoenfeld-residual-based diagnostics; because possible time-dependent effects were observed for some categorical covariates, interval-specific Cox models for 0–60 months and >60 months were performed as sensitivity analyses. Metachronous-neoplasm-free survival was estimated using the Kaplan–Meier method, and cumulative probability was expressed as 1 minus the Kaplan–Meier survival estimate. Complete all-cause mortality could not be reliably ascertained in this retrospective dataset; therefore, a formal competing-risk analysis for all-cause death was not feasible. Gastric cancer-specific mortality was available and was recorded in four patients. The previously reported logistic regression analyses comparing recurrence groups with patients with recurrence-free for at least 120 months were retained as secondary exploratory analyses. In these secondary analyses, variables with *P* < 0.05 in univariate comparisons were considered candidates for multivariable logistic regression, with correlated, redundant, or sparse variables excluded to reduce overfitting and collinearity. A two-sided *P* value < 0.05 was considered statistically significant.

### 2.6 Ethical considerations

The study protocol was approved by the Institutional Review Board of Korea University Guro Hospital [IRB No. 2023GR0458]. In our previously published retrospective ESD cohort, informed consent was waived because of the retrospective study design and the study was conducted in accordance with the Declaration of Helsinki; the present study was conducted under the same ethical principles.

## 3 Results

A total of 1,200 patients achieved curative resection and were followed for at least 12 months. The source population substantially overlapped with our previous study (10): 1,048 of the 1,200 patients were included in the previous cohort. During follow-up, 185 patients (15.4%) developed a first metachronous gastric neoplasm and 1,015 remained recurrence-free and were censored at their last documented follow-up. Among the 185 patients with metachronous neoplasms, 102 developed metachronous cancer and 83 had metachronous adenoma only. Among the 102 patients with metachronous gastric cancer, 113 metachronous cancers were identified: 92 patients developed one cancer, 9 developed two, and 1 developed three. Of the 113 lesions, 106 (93.8%) were stage IA, including 94 (83.2%) T1aN0M0 and 12 (10.6%) T1bN0M0 lesions. The remaining seven lesions comprised one stage IB, three stage IIIA, and three stage IIIB cancers. Four gastric cancer-specific deaths were documented during follow-up; complete all-cause mortality was not available. Baseline characteristics of the entire eligible cohort are summarized descriptively in Table 1 without recurrence-group hypothesis testing.

**Table 1:** Baseline characteristics of the entire eligible cohort.

Characteristic	Entire cohort
Patients, n	1,200
Age, years, mean ± SD	64.5 ± 9.4
Male sex, *n* (%)	891 (74.3)
Diabetes mellitus, *n* (%)	261 (21.8)
NLR, median [IQR]	1.69 [1.31–2.22]
Serum albumin, g/dL, mean ± SD	4.23 ± 0.28
Severe endoscopic atrophy (O2-3), *n* (%)	676/1,196 (56.5)
Differentiated histology, *n* (%)	1,087 (90.6)
Persistent *H. pylori* infection, *n* (%)	55/1,162 (4.7)
Synchronous neoplasm, *n* (%)	166 (13.8)
Metachronous neoplasm during follow-up, *n* (%)	185 (15.4)

Values are descriptive for the entire eligible cohort. Percentages for variables with missing data use the available denominator.

SD, standard deviation; IQR, interquartile range; NLR, neutrophil-to-lymphocyte ratio; *H. pylori*, *Helicobacter pylori*.

### 3.1 Primary full-cohort time-to-event analysis

In the clinically specified multivariable Cox model, 1,155 patients with complete covariate data contributed 180 metachronous-neoplasm events. Older age was independently associated with a higher hazard of metachronous neoplasm (adjusted HR: 1.035 per year, 95% CI: 1.015–1.055, *P* < 0.001). Persistent *H. pylori* infection was also independently associated with recurrence (adjusted HR: 2.391, 95% CI: 1.398–4.088, *P* = 0.001). Diabetes mellitus showed a borderline association (adjusted HR: 1.391, 95% CI: 0.988-1.958, *P* = 0.058), whereas male sex, NLR, serum albumin, severe endoscopic atrophy, differentiated histology, and synchronous neoplasm were not independently associated with the endpoint (Table 2). The model contained nine fitted covariate parameters for 180 events (20 events per parameter). Schoenfeld-residual-based diagnostics suggested possible time-dependent effects for some categorical covariates; therefore, interval-specific analyses were performed as sensitivity analyses. Age remained associated with metachronous recurrence in both follow-up intervals, whereas estimates for other covariates varied between intervals (Supplementary Table S1).

**Table 2:** Multivariable cox proportional hazards analysis for first metachronous neoplasm in the entire cohort.

Variable	Adjusted HR	95% CI	*P*-value
Age (per year)	1.035	1.015–1.055	<0.001
Male sex	1.279	0.883–1.852	0.194
Diabetes mellitus	1.391	0.988–1.958	0.058
NLR (continuous)	1.103	0.960–1.267	0.166
Serum albumin (g/dL)	0.813	0.452–1.461	0.489
Severe endoscopic atrophy (O2–3)	1.206	0.880–1.654	0.244
Differentiated histology	1.783	0.870–3.656	0.114
Persistent *H. pylori* infection	2.391	1.398–4.088	0.001
Synchronous neoplasm	0.996	0.639–1.551	0.985

Complete-case model: *n* = 1,155; 180 events; 9 fitted covariate parameters (20 events per parameter).

HR, hazard ratio; CI, confidence interval; NLR, neutrophil-to-lymphocyte ratio; *H. pylori, Helicobacter pylori*.

Kaplan–Meier analysis of the entire cohort showed estimated cumulative probability of metachronous neoplasm of 7.6% at 36 months and 12.3% at 60 months after ESD (Figure 2). The estimated cumulative probability at 120 months was 36.3%; however, only 66 patients remained at risk at that time point, and this long-term estimate should therefore be interpreted cautiously.

**Figure 2:**
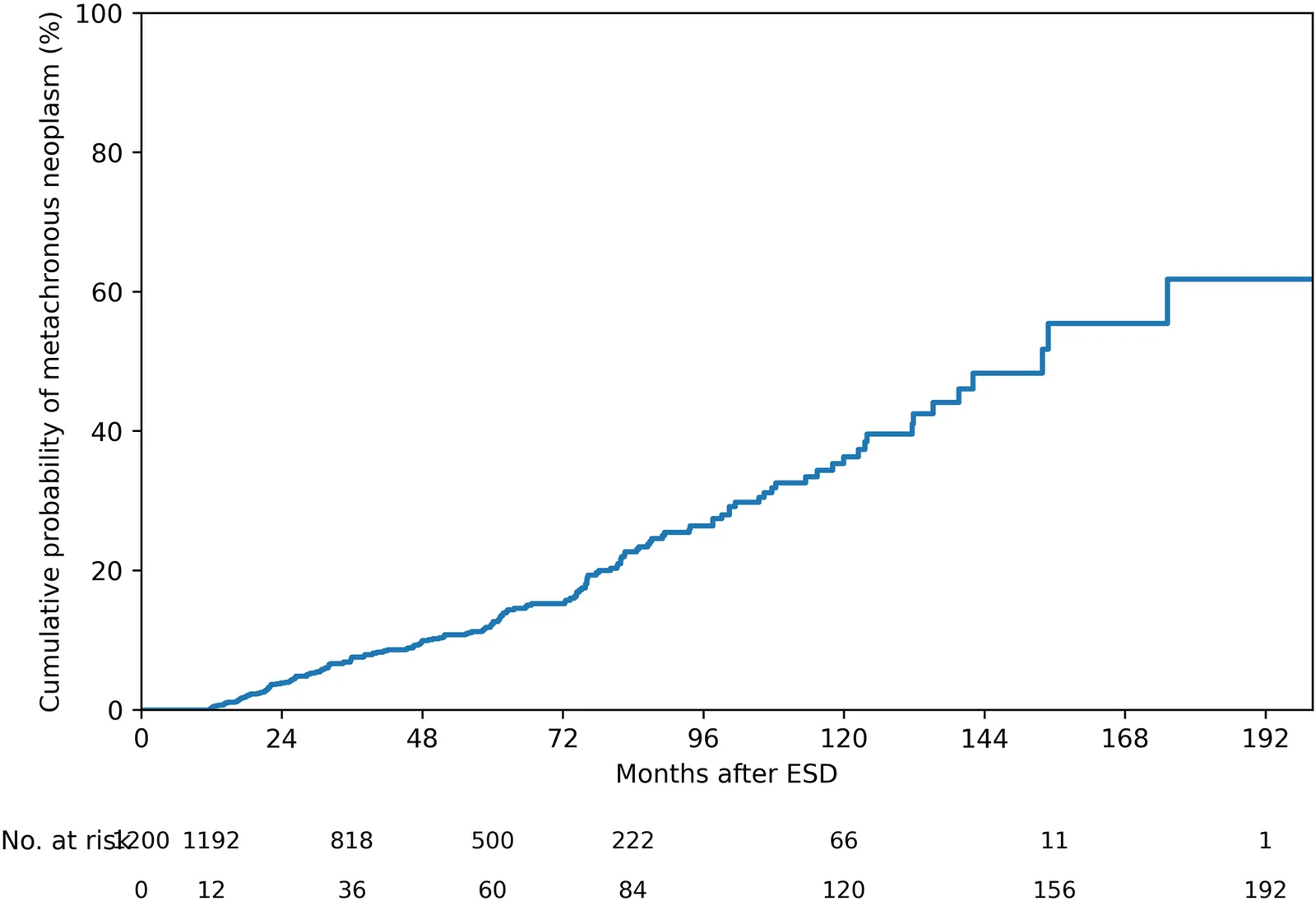
Kaplan–meier estimate of cumulative metachronous-neoplasm probability after curative endoscopic submucosal dissection in the entire cohort. The probability of first metachronous gastric neoplasm was estimated as 1 minus the Kaplan–Meier recurrence-free survival estimate. Numbers at risk are shown below the graph.

### 3.2 Secondary selected-control analyses

As a secondary exploratory analysis, the 185 patients who developed metachronous neoplasms were compared with 70 patients who remained recurrence-free for at least 120 months. The recurrence group was older and showed differences in several clinical, inflammatory, nutritional, metabolic, and clinicopathologic variables compared with the selected long-term recurrence-free controls (Supplementary Tables S2, S3). In multivariable logistic regression, age (OR: 1.091, 95% CI: 1.047–1.137, *P* < 0.001) and diabetes mellitus (OR: 3.636, 95% CI: 1.142–11.573, *P* = 0.029) were associated with metachronous neoplasm (Supplementary Table S4). Because the control group was selected on the basis of future long-term recurrence-free status, these comparisons were considered secondary exploratory analyses rather than estimates of recurrence risk in the overall cohort.

### 3.3 Secondary exploratory analyses of metachronous cancer and adenoma

In cancer-specific selected-control analyses, 102 patients with metachronous cancer were compared with the same 70 long-term recurrence-free controls. Age and diabetes mellitus were associated with metachronous cancer in the multivariable logistic model (age: OR 1.109, 95% CI 1.058–1.163, *P* < 0.001; diabetes mellitus: OR 3.471, 95% CI 1.001–12.032, *P* = 0.049). Baseline, clinicopathologic, and regression results are provided in Supplementary Tables S5–S7. These findings are exploratory and do not demonstrate that the association of diabetes differs between cancer and adenoma in the absence of a formal interaction test.

In the adenoma-only selected-control analysis, 83 patients with metachronous adenoma only were compared with the 70 selected controls. Age (OR: 1.086, 95% CI: 1.035–1.140, *P* = 0.001) and lower serum albumin (OR: 0.134, 95% CI: 0.026–0.686, *P* = 0.016) were associated with metachronous adenoma in the multivariable logistic model. Detailed baseline, clinicopathologic, and regression results are provided in Supplementary Tables S8–S10. Because adenoma-only status depends on the absence of subsequent metachronous cancer during observed follow-up, this analysis was not used as a primary time-to-event endpoint and was considered exploratory.

## 4 Discussion

In this retrospective study, the primary analysis used the complete cohort of 1,200 patients in a time-to-event framework. The principal finding was that older age and persistent *H. pylori* infection were independently associated with a higher hazard of first metachronous gastric neoplasm after curative ESD. Diabetes mellitus, which had been significant in the selected-control logistic analysis, showed only a borderline association in the adjusted full-cohort Cox model. In the secondary analyses using patients who remained recurrence-free for ≥10 years as controls, age and diabetes mellitus were associated with overall metachronous neoplasm and cancer, whereas age and lower serum albumin were associated with metachronous adenoma. These differences may partly reflect the use of individual follow-up time and appropriate censoring in the full-cohort analysis. The cancer and adenoma selected-control analyses were therefore retained only as exploratory secondary analyses and were not used to infer biologically distinct recurrence mechanisms.

The association between older age and metachronous neoplasm is clinically plausible and consistent with the overall conceptual framework of gastric carcinogenesis after endoscopic resection. Because ESD preserves the stomach, the remnant gastric mucosa remains exposed to chronic inflammation, atrophic gastritis, intestinal metaplasia, and field cancerization (19, 20). In our previous ESD study comparing younger (age ≤50 years) and older (age >50 years) patients, older age was also associated with a higher incidence of metachronous gastric neoplasm during long-term follow-up (10). The present analysis extends that observation by using the complete eligible cohort and explicitly incorporating time at risk and censoring. The source population substantially overlaps with our previous study; however, the current study addresses a different question by evaluating a broader set of baseline clinical and laboratory variables in relation to time to first metachronous neoplasm.

Persistent *H. pylori* infection was independently associated with metachronous neoplasm in the primary Cox model. This finding is consistent with the established role of *H. pylori*-driven chronic inflammation and field carcinogenesis in the remnant stomach and with evidence that eradication reduces, but does not eliminate, the risk of metachronous gastric cancer (21). Because the number of patients with persistent infection was relatively small and the proportional-hazards diagnostics suggested possible time-dependent effect, the estimated HR should be interpreted cautiously. Nevertheless, the full-cohort analysis supports persistent infection as a clinically relevant marker that warrants attention during post-ESD follow-up.

Diabetes mellitus illustrates why the full-cohort analysis changes the interpretation of this study. Diabetes is associated with insulin resistance, hyperinsulinemia, chronic inflammation, oxidative stress, and altered insulin-like growth factor signaling, all of which may promote carcinogenesis (22, 23). In this study, diabetes was associated with metachronous recurrence in the selected-control logistic analysis, but its adjusted association in the primary Cox model was borderline (HR: 1.391, 95% CI: 0.988–1.958, *P* = 0.058). Accordingly, the present data do not establish diabetes as an independent determinant of metachronous recurrence. In the secondary analyses, diabetes mellitus showed a possible association with metachronous neoplasm; however, this finding remains exploratory and requires confirmation in larger cohorts.

Continuous NLR and serum albumin were not independently associated with recurrence in the primary full-cohort Cox model. Although lower albumin showed associations in some subgroup analyses, these analyses used selected comparison groups and are therefore exploratory. These markers may reflect systemic inflammatory or nutritional status (24–26), but the present study does not establish them as independent time-to-event predictors of metachronous recurrence. Tumor size, differentiated histology, and several laboratory variables differed between recurrence groups and the highly selected long-term controls, but such contrasts may be amplified by the control-selection strategy. These findings support interpreting the selected-control comparisons as descriptive and exploratory rather than as the primary basis for recurrence-risk estimation. Accordingly, the detailed selected-control baseline and logistic regression tables are presented as Supplementary Tables.

The present study has two main clinical implications. First, the full-cohort time-to-event analysis demonstrates that metachronous recurrence continues to accumulate during long-term follow-up; the estimated cumulative probability was 7.6% at 3 years and 12.3% at 5 years. The 10-year estimate was higher, but relatively few patients remained at risk at that time and the estimate should be interpreted cautiously. Second, 93.8% of metachronous cancers were detected at stage IA, supporting continued endoscopic surveillance after curative ESD. The present findings are not intended to justify omission or reduction of surveillance or to define a validated risk-stratified surveillance schedule. Rather, age and persistent *H. pylori* infection may help characterize patients with differing recurrence hazards and should be evaluated prospectively before clinical implementation.

Several limitations should be acknowledged. First, this was a retrospective single-center study and remains susceptible to residual confounding and information bias. Second, although the primary analysis included the entire eligible cohort and accounted for variable follow-up and censoring, complete all-cause mortality could not be reliably ascertained; therefore, death could not be formally treated as a competing event. Four gastric cancer-specific deaths were documented, but this does not substitute for complete all-cause mortality data. Third, some covariates had missing values, and the adjusted Cox model therefore used 1,155 complete cases with 180 events. Fourth, Schoenfeld-residual-based diagnostics suggested possible non-proportional hazards for some categorical covariates; interval-specific Cox analyses were therefore performed as sensitivity analyses, but time-varying estimates should be interpreted cautiously. Fifth, histologic staging of background gastric mucosal changes using OLGIM was unavailable because systematic mapping was not routinely performed during the study period. Sixth, the secondary selected-control analyses used patients recurrence-free for at least 120 months and may exaggerate between-group contrasts; these analyses were retained only as secondary exploratory analyses. Finally, multiple secondary comparisons increase the possibility of chance findings, and external validation is required.

## 5 Conclusion

Despite these limitations, this study has the strength of analyzing all 1,200 eligible patients in the primary time-to-event model. This approach uses the available follow-up information from the entire cohort and appropriately accounts for censoring. The study also provides detailed characterization of metachronous cancers and retains the selected-control analyses as clearly labeled exploratory analyses for comparison.

In conclusion, older age and persistent *H. pylori* infection were independently associated with a higher hazard of metachronous gastric neoplasm after curative ESD for EGC in the full cohort, whereas the association with diabetes mellitus was borderline after adjustment. Secondary cancer- and adenoma-specific selected-control analyses were regarded as exploratory and do not establish different biological determinants between recurrence subtypes. Continued endoscopic surveillance remains necessary after curative ESD, and the potential clinical value of the identified factors requires validation in independent prospective cohorts.

## Data Availability

The raw data supporting the conclusions of this article will be made available by the authors, without undue reservation.
