# Supplementary material for: Clinical parameters associated with metachronous recurrence after curative endoscopic submucosal dissection for early gastric cancer
Source: Front Med (Lausanne). 2026 Oct 23;13:1965395. doi: 10.3389/fmed.2026.1965395 (PMC13739508; doi:10.3389/fmed.2026.1965395)
Supplement: Supplementary file 1 [file Table1.docx]

**Supplementary Table S1. Sensitivity analysis using interval-specific Cox proportional hazards models**

| **Variable** | **0–60 months HR (95% CI)** | ***P*-value** | **>60 months HR (95% CI)** | ***P*-value** |
| --- | --- | --- | --- | --- |
| Age, per year | 1.031 (1.006–1.056) | 0.014 | 1.052 (1.018–1.086) | 0.002 |
| Male sex | 1.120 (0.704–1.783) | 0.633 | 1.615 (0.867–3.008) | 0.131 |
| Diabetes mellitus | 1.223 (0.795–1.881) | 0.361 | 1.736 (0.994–3.031) | 0.052 |
| NLR | 1.028 (0.832–1.270) | 0.796 | 1.194 (1.003–1.421) | 0.046 |
| Albumin | 0.514 (0.256–1.031) | 0.061 | 2.368 (0.846–6.628) | 0.101 |
| Severe atrophy | 1.287 (0.854–1.939) | 0.228 | 0.990 (0.603–1.624) | 0.969 |
| Differentiated histology | 9.198 (1.278–66.188) | 0.028 | 0.671 (0.295–1.524) | 0.340 |
| Persistent *H. pylori* | 1.882 (0.931–3.804) | 0.078 | 3.685 (1.588–8.548) | 0.002 |
| Synchronous neoplasm | 0.846 (0.481–1.490) | 0.562 | 1.365 (0.668–2.788) | 0.393 |

HR, hazard ratio; CI, confidence interval; *H. pylori, Helicobacter pylori*

**Supplementary Table S2. Baseline characteristics of patients with metachronous neoplasm and selected long-term recurrence-free controls**

|  | **Metachronous neoplasm group**  **(n=185)** | **No recurrence Group**  **(n=70)** | ***P*-value** |
| --- | --- | --- | --- |
| Age (years ± SD) | 65.8 ± 8.2 | 58.3 ±8.4 | <0.001 |
| Gender (male: female) | 145 (78.4%): 40 (21.6%) | 51 (72.9%): 19 (27.1%) | 0.351 |
| Family history of GC (n, %) | 18 (9.7%) | 6 (8.6%) | 0.777 |
| Smoking (n, %) |  |  | 0.175 |
| Never smoking | 107 (57.8%) | 47 (67.1%) |  |
| Past or current smoker | 78 (42.2%) | 23 (32.9%) |  |
| Comorbidity (n, %) |  |  |  |
| Hypertension | 92 (49.7%) | 25 (35.7%) | 0.045 |
| Diabetes mellitus | 49 (26.5%) | 5 (7.1%) | <0.001 |
| Heart disease | 18 (9.7%) | 4 (5.7%) | 0.308 |
| Cerebrovascular accident | 9 (4.9%) | 2 (2.9%) | 0.732 |
| End-stage renal disease | 1 (0.5%) | 0 (0.0%) | 1.000 |
| Height (m ± SD) | 1.64 ± 0.08 | 1.64 ± 0.07 | 0.484 |
| Weight (kg ± SD) | 66.6 ± 9.9 | 66.3 ± 10.2 | 0.500 |
| BMI (kg/m^2^ ± SD) | 24.5 ±3.1 | 24.6 ±3.1 | 0.235 |
| WBC count (/µL, median [IQR]) | 6500 [5600–7700] | 5850 [5100–7175] | 0.016 |
| Neutrophil count (/µL, median [IQR]) | 3636.2 [3029–4468] | 3089.8 [2601–3834] | <0.001 |
| Lymphocyte count (/µL, median [IQR]) | 2073.0 [1679–2469] | 2108.2 [1763–2591] | 0.288 |
| Platelet count (×10³/µL ± SD) | 217.9 ± 55.9 | 219.3 ± 57.8 | 0.860 |
| NLR (median [IQR]) | 1.81 [1.32–2.34] | 1.49 [1.09–1.86] | <0.001 |
| PLR (median [IQR]) | 101.1 [80.2–127.8] | 98.4 [81.5–133.6] | 0.548 |
| ESR (mm/hr, median [IQR]) | 12 [6.8–22] | 12 [7–19] | 0.951 |
| Albumin (g/dL ± SD) | 4.20 ± 0.26 | 4.29 ± 0.23 | 0.011 |
| PNI (± SD) | 52.8 ± 5.0 | 54.2 ± 4.9 | 0.039 |
| Total cholesterol (mg/dL ± SD) | 172.1 ± 36.1 | 185.6 ± 38.5 | 0.013 |
| HDL (mg/dL± SD) | 48.6 ± 13.1 | 48.8 ± 10.8 | 0.941 |
| LDL (mg/dL ± SD) | 98.7 ± 34.0 | 109.5 ± 33.9 | 0.029 |
| TG (mg/dL, median [IQR]) | 129 [86–183] | 125 [85.3–179] | 0.772 |
| Fasting glucose (mg/dL, median [IQR]) | 105 [95–128] | 98.5 [92–107] | 0.008 |
| TGI (± SD) | 8.86 ± 0.66 | 8.78 ± 0.62 | 0.355 |
| Serum creatinine (mg/dL, median [IQR]) | 0.82 [0.71–0.92] | 0.79 [0.69–0.90] | 0.195 |
| CRP (mg/dL, median [IQR]) | 0.75 [0.37–1.65] | 0.55 [0.38–1.20] | 0.200 |

Note: SD, standard deviation; GC, gastric cancer; *H. pylori, Helicobacter pylori*; BMI, body mass index; WBC, white blood cell; IQR, interquartile range; NLR, neutrophil-to-lymphocyte ratio; PLR, platelet-to-lymphocyte ratio; ESR, erythrocyte sediment rate; PNI, prognostic nutritional index; HDL, high-density lipoprotein; LDL, low-density lipoprotein; TG, triglyceride; TGI, triglyceride-glucose index; CRP, C-reactive protein

**Supplementary Table S3. Clinicopathologic characteristics of patients with metachronous neoplasm and selected long-term recurrence-free controls**

|  | **Metachronous neoplasm group**  **(n=185)** | **No recurrence group**  **(n=70)** | ***P*-value** |
| --- | --- | --- | --- |
| Lesion location (n, %) |  |  | 0.201 |
| Lower third | 117 (63.2%) | 44 (62.9%) |  |
| Mid third | 60 (34.5%) | 19 (27.1%) |  |
| Upper third | 8 (4.3%) | 7 (10.0%) |  |
| Lesion shape (n, %) |  |  | 0.388 |
| Elevated (I, IIa) | 52 (28.1%) | 13 (18.6%) |  |
| Flat (IIb) | 68 (36.8%) | 31 (44.3%) |  |
| Depressed (IIc) | 65 (35.1%) | 26 (37.1%) |  |
| Presence of ulcer (n, %) | 19 (10.3%) | 5 (7.1%) | 0.445 |
| Tumor size (mm, median [IQR]) | 16 [8–23] | 12 [8–18] | 0.045 |
| Atrophy (n, %) |  |  | 0.507 |
| C1-2 | 5 (2.7%) | 3 (4.3%) |  |
| C3-O1 | 60 (32.4%) | 26 (37.1%) |  |
| O2-3 | 120 (64.9%) | 41 (58.6%) |  |
| Post-ESD diagnosis (n, %) |  |  | 0.081 |
| WD adenocarcinoma | 104 (56.2%) | 39 (55.7%) |  |
| MD adenocarcinoma | 73 (39.5%) | 22 (31.4%) |  |
| PD adenocarcinoma | 5 (2.7%) | 5 (7.1%) |  |
| SRC | 3 (1.6%) | 4 (5.7%) |  |
| Differentiation (n, %) |  |  | 0.023 |
| Differentiated type | 177 (95.7%) | 61 (87.1%) |  |
| Undifferentiated type | 8 (4.3%) | 9 (12.9%) |  |
| Lauren classification type (n, %) |  |  | 0.033 |
| Intestinal | 178 (96.2%) | 62 (88.6%) |  |
| Diffuse or mixed | 7 (3.7%) | 8 (11.3%) |  |
| Depth of invasion (n, %) |  |  | 0.067 |
| Mucosa | 174 (94.1%) | 61 (87.1%) |  |
| Submucosa | 11 (5.9%) | 9 (12.9%) |  |
| Criteria (n, %) |  |  | 0.301 |
| Absolute | 87 (47.0%) | 38 (54.3%) |  |
| Expanded | 98 (53.0%) | 32 (45.7%) |  |
| *H. pylori* infection (n, %) |  |  | 0.047 |
| Negative or eradicated | 165 (91.2%) | 68 (98.6%) |  |
| Persistent | 16 (8.8%) | 1 (1.4%) |  |
| Synchronous neoplasm (n, %) | 24 (13.0%) | 5 (7.1%) | 0.191 |
| Follow-up duration (months, median [IQR]) | 86.9 [55.9–121.1] | 129.4 [120.5–145.2] | <0.001 |

Note: IQR, interquartile range; WD, well differentiated; MD, moderately differentiated; PD, poorly differentiated; SRC, signet ring cell carcinoma; *H. pylori, Helicobacter pylori*

**Supplementary Table S4 Multivariable logistic regression analysis for metachronous neoplasm in the selected-control analysis**

| **Variables** | **Multivariate analysis** | | |
| --- | --- | --- | --- |
|  | **OR** | **95% CI** | **P-value** |
| Age (years) | 1.091 | 1.047 – 1.137 | <0.001 |
| Hypertension | 1.107 | 0.575 – 2.130 | 0.761 |
| Diabetes mellitus | 3.636 | 1.142 – 11.573 | 0.029 |
| NLR | 1.461 | 0.920 – 2.318 | 0.108 |
| Serum albumin (g/dL) | 0.387 | 0.103 – 1.459 | 0.161 |
| Fasting glucose (mg/dL) | 1.004 | 0.991 – 1.017 | 0.586 |
| Differentiated type | 1.615 | 0.505 – 5.167 | 0.419 |
| Tumor size (mm) | 1.023 | 0.990 – 1.057 | 0.168 |

OR, odds ratio; CI, confidence interval; NLR, neutrophil-to-lymphocyte ratio

**Supplementary Table S5 Baseline characteristics of patients with metachronous cancer and selected long-term recurrence-free controls**

|  | **Metachronous cancer group**  **(n=102)** | **No recurrence group**  **(n=70)** | ***P*-value** |
| --- | --- | --- | --- |
| Age (years ± SD) | 66.5 ± 8.4 | 58.3 ± 8.4 | <0.001 |
| Gender (male: female) | 84 (82.4%): 18 (17.6%) | 51 (72.9%): 19 (27.1%) | 0.137 |
| Family history of GC (n, %) | 13 (12.7%) | 6 (8.6%) | 0.391 |
| Smoking (n, %) |  |  | 0.218 |
| Never smoking | 59 (57.8%) | 47 (67.1%) |  |
| Past or current smoker | 43 (42.2%) | 23 (32.9%) |  |
| Comorbidity (n, %) |  |  |  |
| Hypertension | 47 (46.1%) | 25 (35.7%) | 0.176 |
| Diabetes mellitus | 28 (27.5%) | 5 (7.1%) | 0.001 |
| Heart disease | 11 (10.8%) | 4 (5.7%) | 0.247 |
| Cerebrovascular accident | 4 (3.9%) | 2 (2.9%) | 1.000 |
| End-stage renal disease | 0 (0.0%) | 0 (0.0%) |  |
| Height (m ± SD) | 1.65 ± 0.07 | 1.64 ± 0.07 | 0.702 |
| Weight (kg ± SD) | 65.9 ± 9.0 | 66.3 ± 10.2 | 0.789 |
| BMI (kg/m^2^ ± SD) | 24.3 ± 2.7 | 24.6 ±3.1 | 0.568 |
| WBC count (/µL, median [IQR]) | 6800 [5800–7900] | 5850 [5100–7175] | 0.002 |
| Neutrophil count (/µL, median [IQR]) | 3781.2 [3277–4686] | 3089.8 [2601–3834] | <0.001 |
| Lymphocyte count (/µL, median [IQR]) | 2104.9 [1584–2528] | 2108.2 [1763–2591] | 0.461 |
| Platelet count (×10³/µL ± SD) | 212.1 ± 57.3 | 219.3 ± 57.8 | 0.420 |
| NLR (median [IQR]) | 1.85 [1.36–2.51] | 1.49 [1.09–1.86] | <0.001 |
| PLR (median [IQR]) | 98.2 [77.3–125.3] | 98.4 [81.5–133.6] | 0.926 |
| ESR (mm/hr, median [IQR]) | 12 [6–22] | 12 [7–19] | 0.949 |
| Albumin (g/dL ± SD) | 4.23 ± 0.26 | 4.29 ± 0.23 | 0.130 |
| PNI (± SD) | 53.3 ± 5.2 | 54.2 ± 4.9 | 0.220 |
| Total cholesterol (mg/dL ± SD) | 171.3 ± 35.4 | 185.6 ± 38.5 | 0.016 |
| HDL (mg/dL± SD) | 49.6 ± 13.9 | 48.8 ± 10.8 | 0.650 |
| LDL (mg/dL ± SD) | 97.6 ± 33.9 | 109.5 ± 33.9 | 0.031 |
| TG (mg/dL, median [IQR]) | 121 [84–183] | 125 [85.3–179] | 0.861 |
| Fasting glucose (mg/dL, median [IQR]) | 104 [95.5–122.5] | 98.5 [92–107] | 0.023 |
| TGI (± SD) | 8.82 ± 0.68 | 8.78 ± 0.62 | 0.664 |
| Serum creatinine (mg/dL, median [IQR]) | 0.82 [0.73–0.91] | 0.79 [0.69–0.90] | 0.136 |
| CRP (mg/dL, median [IQR]) | 0.75 [0.39–2.00] | 0.55 [0.38–1.20] | 0.096 |

Note: SD, standard deviation; GC, gastric cancer; *H. pylori, Helicobacter pylori*; BMI, body mass index; WBC, white blood cell; IQR, interquartile range; NLR, neutrophil-to-lymphocyte ratio; PLR, platelet-to-lymphocyte ratio; ESR, erythrocyte sediment rate; PNI, prognostic nutritional index; HDL, high-density lipoprotein; LDL, low-density lipoprotein; TG, triglyceride; TGI, triglyceride-glucose index; CRP, C-reactive protein

**Supplementary Table S6 Clinicopathologic characteristics of patients with metachronous cancer and selected long-term recurrence-free controls**

|  | **Metachronous cancer group**  **(n=102)** | **No recurrence group**  **(n=70)** | ***P*-value** |
| --- | --- | --- | --- |
| Lesion location (n, %) |  |  | 0.281 |
| Lower third | 61 (58.9%) | 44 (62.9%) |  |
| Mid third | 36 (35.3%) | 19 (27.1%) |  |
| Upper third | 5 (4.9%) | 7 (10.0%) |  |
| Lesion shape (n, %) |  |  | 0.409 |
| Elevated (I, IIa) | 27 (26.5%) | 13 (18.6%) |  |
| Flat (IIb) | 37 (36.3%) | 31 (44.3%) |  |
| Depressed (IIc) | 38 (37.3%) | 26 (37.1%) |  |
| Presence of ulcer (n, %) | 11 (10.8%) | 5 (7.1%) | 0.419 |
| Tumor size (mm, median [IQR]) | 14 [10–23] | 12 [8–18] | 0.082 |
| Atrophy (n, %) |  |  | 0.383 |
| C1-2 | 2 (2.0%) | 3 (4.3%) |  |
| C3-O1 | 31 (30.4%) | 26 (37.1%) |  |
| O2-3 | 69 (67.6%) | 41 (58.6%) |  |
| Post-ESD diagnosis (n, %) |  |  | 0.161 |
| WD adenocarcinoma | 55 (53.9%) | 39 (55.7%) |  |
| MD adenocarcinoma | 42 (41.2%) | 22 (31.4%) |  |
| PD adenocarcinoma | 4 (3.9%) | 5 (7.1%) |  |
| SRC | 1 (1.0%) | 4 (5.7%) |  |
| Differentiation (n, %) |  |  | 0.061 |
| Differentiated type | 97 (95.1%) | 61 (87.1%) |  |
| Undifferentiated type | 5 (4.9%) | 9 (12.9%) |  |
| Lauren classification type (n, %) |  |  | 0.071 |
| Intestinal | 98 (96.1%) | 62 (88.6%) |  |
| Diffuse or mixed | 4 (3.9%) | 8 (11.3%) |  |
| Depth of invasion (n, %) |  |  | 0.184 |
| Mucosa | 95 (93.1%) | 61 (87.1%) |  |
| Submucosa | 7 (6.9%) | 9 (12.9%) |  |
| Criteria (n, %) |  |  | 0.581 |
| Absolute | 51 (50.0%) | 38 (54.3%) |  |
| Expanded | 51 (50.0%) | 32 (45.7%) |  |
| *H. pylori* infection (n, %) |  |  | 0.142 |
| Negative or eradicated | 91 (92.9%) | 68 (98.6%) |  |
| Persistent | 7 (7.1%) | 1 (1.4%) |  |
| Synchronous neoplasm (n, %) | 15 (14.7%) | 5 (7.1%) | 0.128 |
| Follow-up duration (months, median [IQR]) | 92.5 [56.3–129.1] | 129.4 [120.5–145.2] | <0.001 |

Note: IQR, interquartile range; WD, well differentiated; MD, moderately differentiated; PD, poorly differentiated; SRC, signet ring cell carcinoma; *H. pylori, Helicobacter pylori*

**Supplementary Table S7 Multivariable logistic regression analysis for metachronous cancer in the selected-control analysis**

| **Variables** | **Multivariate analysis** | | |
| --- | --- | --- | --- |
|  | **OR** | **95% CI** | **P-value** |
| Age (years) | 1.109 | 1.058 – 1.163 | <0.001 |
| Diabetes mellitus | 3.471 | 1.001 – 12.032 | 0.049 |
| NLR | 1.563 | 0.947 – 2.579 | 0.080 |
| Fasting glucose (mg/dL) | 1.001 | 0.988 – 1.014 | 0.875 |
| Differentiated type | 1.905 | 0.484 – 7.498 | 0.356 |

OR, odds ratio; CI, confidence interval; NLR, neutrophil-to-lymphocyte ratio

**Supplementary Table S8 Baseline characteristics of patients with metachronous adenoma only and selected long-term recurrence-free controls**

|  | **Metachronous adenoma group**  **(n=83)** | **No recurrence group**  **(n=70)** | ***P*-value** |
| --- | --- | --- | --- |
| Age (years ± SD) | 64.9 ± 8.2 | 58.3 ± 8.4 | <0.001 |
| Gender (male: female) | 61 (73.5%): 22 (26.5%) | 51 (72.9%): 19 (27.1%) | 0.929 |
| Family history of GC (n, %) | 5 (6.0%) | 6 (8.6%) | 0.543 |
| Smoking (n, %) |  |  | 0.237 |
| Never smoking | 48 (57.8%) | 47 (67.1%) |  |
| Past or current smoker | 35 (42.2%) | 23 (32.9%) |  |
| Comorbidity (n, %) |  |  |  |
| Hypertension | 45 (54.2%) | 25 (35.7%) | 0.022 |
| Diabetes mellitus | 21 (25.3%) | 5 (7.1%) | 0.003 |
| Heart disease | 7 (8.4%) | 4 (5.7%) | 0.517 |
| Cerebrovascular accident | 5 (6.0%) | 2 (2.9%) | 0.454 |
| End-stage renal disease | 1 (1.2%) | 0 (0.0%) | 1.000 |
| Height (m ± SD) | 1.62 ± 0.08 | 1.64 ± 0.07 | 0.193 |
| Weight (kg ± SD) | 65.2 ± 10.9 | 66.3 ± 10.2 | 0.528 |
| BMI (kg/m^2^ ± SD) | 24.7 ±3.5 | 24.6 ±3.1 | 0.864 |
| WBC count (/µL, median [IQR]) | 6100 [5400–7400] | 5850 [5100–7175] | 0.357 |
| Neutrophil count (/µL, median [IQR]) | 3451.5 [2890–4271] | 3089.8 [2601–3834] | 0.055 |
| Lymphocyte count (/µL, median [IQR]) | 2046 [1695–2402] | 2108.2 [1763–2591] | 0.240 |
| Platelet count (×10³/µL ± SD) | 225.0 ± 53.7 | 219.3 ± 57.8 | 0.535 |
| NLR (median [IQR]) | 1.78 [1.30–2.21] | 1.49 [1.09–1.86] | 0.010 |
| PLR (median [IQR]) | 113.9 [83.7–134.8] | 98.4 [81.5–133.6] | 0.207 |
| ESR (mm/hr, median [IQR]) | 12 [7–20.5] | 12 [7–19] | 0.968 |
| Albumin (g/dL ± SD) | 4.17 ± 0.26 | 4.29 ± 0.23 | 0.002 |
| PNI (± SD) | 52.2 ± 4.6 | 54.2 ± 4.9 | 0.010 |
| Total cholesterol (mg/dL ± SD) | 173.1 ± 37.0 | 185.6 ± 38.5 | 0.046 |
| HDL (mg/dL± SD) | 47.4 ± 12.0 | 48.8 ± 10.8 | 0.473 |
| LDL (mg/dL ± SD) | 100.0 ± 34.4 | 109.5 ± 33.9 | 0.099 |
| TG (mg/dL, median [IQR]) | 137 [94–180] | 125 [85.3–179] | 0.446 |
| Fasting glucose (mg/dL, median [IQR]) | 105 [95.3–136.8] | 98.5 [92–107] | 0.014 |
| TGI (± SD) | 8.91 ± 0.63 | 8.78 ± 0.62 | 0.193 |
| Serum creatinine (mg/dL, median [IQR]) | 0.81 [0.68–0.95] | 0.79 [0.69–0.90] | 0.457 |
| CRP (mg/dL, median [IQR]) | 0.72 [0.32–1.59] | 0.55 [0.38–1.20] | 0.596 |

Note: SD, standard deviation; GC, gastric cancer; *H. pylori, Helicobacter pylori*; BMI, body mass index; WBC, white blood cell; IQR, interquartile range; NLR, neutrophil-to-lymphocyte ratio; PLR, platelet-to-lymphocyte ratio; ESR, erythrocyte sediment rate; PNI, prognostic nutritional index; HDL, high-density lipoprotein; LDL, low-density lipoprotein; TG, triglyceride; TGI, triglyceride-glucose index; CRP, C-reactive protein

**Supplementary Table S9 Clinicopathologic characteristics of patients with metachronous adenoma only and selected long-term recurrence-free controls**

|  | **Metachronous adenoma group**  **(n=83)** | **No recurrence group**  **(n=70)** | ***P*-value** |
| --- | --- | --- | --- |
| Lesion location (n, %) |  |  | 0.309 |
| Lower third | 56 (67.5%) | 44 (62.9%) |  |
| Mid third | 24 (28.9%) | 19 (27.1%) |  |
| Upper third | 3 (3.6%) | 7 (10.0%) |  |
| Lesion shape (n, %) |  |  | 0.294 |
| Elevated (I, IIa) | 26 (31.3%) | 13 (18.6%) |  |
| Flat (IIb) | 31 (37.4%) | 31 (44.3%) |  |
| Depressed (IIc) | 26 (31.3%) | 26 (37.1%) |  |
| Presence of ulcer (n, %) | 8 (9.6%) | 5 (7.1%) | 0.581 |
| Tumor size (mm, median [IQR]) | 16 [8–23] | 12 [8–18] | 0.070 |
| Atrophy (n, %) |  |  | 0.958 |
| C1-2 | 3 (3.6%) | 3 (4.3%) |  |
| C3-O1 | 29 (34.9%) | 26 (37.1%) |  |
| O2-3 | 51 (61.4%) | 41 (58.6%) |  |
| Post-ESD diagnosis (n, %) |  |  | 0.188 |
| WD adenocarcinoma | 49 (59.0%) | 39 (55.7%) |  |
| MD adenocarcinoma | 31 (37.3%) | 22 (31.4%) |  |
| PD adenocarcinoma | 1 (1.2%) | 5 (7.1%) |  |
| SRC | 2 (2.4%) | 4 (5.7%) |  |
| Differentiation (n, %) |  |  | 0.034 |
| Differentiated type | 80 (96.4%) | 61 (87.1%) |  |
| Undifferentiated type | 3 (3.6%) | 9 (12.9%) |  |
| Lauren classification type (n, %) |  |  | 0.149 |
| Intestinal | 80 (96.4%) | 62 (88.6%) |  |
| Diffuse or mixed | 3 (3.6%) | 8 (11.3%) |  |
| Depth of invasion (n, %) |  |  | 0.076 |
| Mucosa | 79 (95.2%) | 61 (87.1%) |  |
| Submucosa | 4 (4.8%) | 9 (12.9%) |  |
| Criteria (n, %) |  |  | 0.178 |
| Absolute | 36 (43.3%) | 38 (54.3%) |  |
| Expanded | 47 (56.7%) | 32 (45.7%) |  |
| *H. pylori* infection (n, %) |  |  | 0.022 |
| Negative or eradicated | 74 (89.2%) | 68 (98.6%) |  |
| Persistent | 9 (10.8%) | 1 (1.4%) |  |
| Synchronous neoplasm (n, %) | 9 (10.8%) | 5 (7.1%) | 0.429 |
| Follow-up durations (month, median [IQR]) | 86.0 [54.9–114.8] | 129.4 [120.5–145.2] | <0.001 |

Note: IQR, interquartile range; WD, well differentiated; MD, moderately differentiated; PD, poorly differentiated; SRC, signet ring cell carcinoma; *H. pylori, Helicobacter pylori*

**Supplementary Table S10. Multivariable logistic regression analysis for metachronous adenoma in the selected-control analysis**

| **Variables** | **Multivariate analysis** | | |
| --- | --- | --- | --- |
|  | **OR** | **95% CI** | **P-value** |
| Age (years) | 1.086 | 1.035 – 1.140 | 0.001 |
| Hypertension | 1.150 | 0.524 – 2.525 | 0.728 |
| Diabetes mellitus | 3.537 | 0.908 – 13.778 | 0.069 |
| NLR | 1.248 | 0.707 – 2.203 | 0.446 |
| Serum albumin (g/dL) | 0.134 | 0.026 – 0.686 | 0.016 |
| Fasting glucose (mg/dL) | 1.005 | 0.990 – 1.019 | 0.522 |
| Total cholesterol (mg/dL) | 0.996 | 0.986 – 1.006 | 0.440 |
| Differentiated type | 1.644 | 0.361 – 7.490 | 0.520 |

OR, odds ratio; CI, confidence interval; NLR, neutrophil-to-lymphocyte ratio;
